# Subjective memory complaints and medication adherence among hypertensive Korean older adults with multimorbidity: mediating effect of depression and social support

**DOI:** 10.1186/s12889-024-18061-4

**Published:** 2024-02-23

**Authors:** Jeong Sun Kim, Eunji Kim

**Affiliations:** 1 College of Nursing, Chonnam National University, 160, Baekseo-ro, Dong-gu, Gwangju, South Korea; 2 Department of Public Health Science, Graduate School of Public Health, Seoul National University, 1 Gwanak-ro, Gwanak-gu, Seoul, South Korea

**Keywords:** Medication adherence, Subjective memory complaints, Depression, Social support

## Abstract

**Background & Aim(s):**

Medication adherence (MA) is a key factor in maintaining adequate blood pressure and preventing complications. However, some older adults experience difficulties in taking medicine properly due to declines in cognitive function. Although subjective memory complaints (SMC) are recognized as early markers of cognitive impairment, previous studies concerning the relationship between MA and cognitive function have focused only on objective cognitive function. Furthermore, while depression has a high correlation with SMC, low MA, and social support, there is limited evidence on their relationship. This study aims to understand the effect of SMC on MA and the mediating effect of depression and social support.

**Method(s):**

This study is a descriptive cross-sectional investigation. A sample of 195 community-dwelling hypertensive older adults with multimorbidity from 3 community senior centers in Gwangju, South Korea were recruited through convenience sampling. Data was collected through face-to-face survey from January to March 2018. The PROCESS macro v4.2 program [Model 6] was used to analyze the mediating effect of depression and social support in the relationship between SMC and MA. Data analysis was performed using SPSS/WIN 26.0 and STATA MP 17.0.

**Results:**

The average MA was 6.74. There were significant differences in MA according to awareness of prescribed drugs, awareness of side effects, insomnia, and healthcare accessibility. SMC was positively correlated with depression, while social support and MA were negatively correlated. While depression was a significant mediator of the effect of SMC on MA, the mediating effect of social support was not significant. The multiple mediation effect of depression and social support was not significant.

**Conclusion:**

The results suggest that medication management of older adults in community settings should be accompanied by a comprehensive health assessment of associated factors. Health professionals should explore strategies to improve memory as well as prevent and alleviate depression to increase MA among hypertensive older adults with multimorbidity.

## Background

Population aging is a global phenomenon as the world’s older adult population aged 65 and older has increased from 6% in 1990 to 9.82% in 2022 [1]. Korea is a country experiencing unprecedented rapid aging, with proportion of older adults growing from 5.1 to 17.5% during the same period [2]. The proportion of older adults is expecting to increase to 20.6% entering into super-aged society by 2025 [3].

Developments in life science technologies have contributed to the extension of life expectancy, accompanied by population aging and increases in prevalence and costs of chronic disease [4]. Meta-analysis of the prevalence of multimorbidity across World Health Organization (WHO) regions have found that 51% of community dwelling older adults over 60 years of age have multiple chronic conditions [5]. In Korea, approximately 73% of older adults over 65 have multimorbidity [6]. Multimorbidity is associated with functional decline, disability, and mortality among older adults [7, 8], and is a key predictor of healthcare utilization and costs [9]. Preventative healthcare policies and services are needed to ensure healthy aging for older adults with multimorbidity.

Chronic disease are conditions that persist over long periods of time, and require regular use of prescription medications throughout the lifetime to manage symptoms and minimize complications [4]. Number of chronic diseases and the number of prescribed medications often increases with age; the average Korean older adult intakes 4.1 medications [6]. In particular, older adults with multiple chronic diseases had significantly higher levels of polypharmacy than those with single chronic diseases [10]. Polypharmacy among older adults is associated with risk of adverse drug reactions, increased treatment costs, and non-adherence to medication [11]. Wang and colleagues [12] found having two or more chronic conditions was a significant predictor of medication non-adherence. Meta-analysis of MA rates among multimorbid older adults showed non-adherence rates of 42.6% [13], demonstrating that continuous medication assessment and management is necessary to improve MA among multimorbid older adults.

Hypertension is the most common comorbidity among those with multimorbidity [14, 15]. According to the WHO, approximately 40% of adults over 25 years of age are diagnosed with hypertension [16]. While hypertension is a major risk factor for stroke, cardiovascular disease, renal failure, and premature death, it is referred to as the ‘silent killer’ due to its asymptomatic early stages [16]. Therefore, medication management is crucial for controlling blood pressure and preventing adverse outcomes among patients with hypertension [16, 17]. However, a meta-analysis of MA in hypertensive patients reported a medication non-adherence rate of 45.2% [18]. Furthermore, hypertensive older adults with multimorbidities were found to be at higher risk of polypharmacy and physical frailty, increasing vulnerability against medication non-adherence and its consequences [19]. Therefore, the identification and analysis of the relationship among factors associated with MA is an essential healthcare priority to improve adherence in hypertensive older adults with multimorbidity.

MA in older adults with chronic disease refers to the voluntary practice of using medications as prescribed or directed through interactions with healthcare providers, having positive expectations of the effectiveness of medication, and consistent intake of the medication through mindful awareness [20]. Causes for poor MA among older adults include not only fear of side effects and financial burden, but cognitive impairments such as forgetfulness and memory problems [21]. Multidomain cognitive functions are required for MA, including memory and attention to remember to take the medications, understanding of prescribed directions to take medicine and their side effects, and decision making for coordinating outpatient appointments and medication schedules with daily routines [22]. This is supported by several studies that report the significant association between cognitive decline and MA [22–25]. However, most MA studies have focused on objective measures of cognitive functioning, failing to examine the relationship between MA and subjective memory complaint (SMC) which is experienced by older adults prior to the onset of more observable decline in cognition.

SMC refers to the self-perceived memory decline and associated discomforts, despite absence of impairment based on objective testing [26, 27]. SMC is directly associated to cognitive functions such as attention, working memory, executive function, and processing speed [28].Approximately 40% of older adults experience age-related memory impairments [29], and such self-perceived forgetfulness in older adults induces fear, embarrassment, and low self-esteem [30]. Moreover, high rates of polypharmacy due to complex health conditions leaves older adults at higher risk of medication-related errors such as non-adherence due to forgetfulness or misuse of medications due to lack of misunderstanding of how to take medication [31]. In particular, previous literature suggests hypertensive patients are at higher risk of experiencing decline in memory [32].

In addition to memory problems, depression has been identified as a major predictor of MA in older adults [33]. Lack of motivation to take medication, social isolation, lowered expectations for effectiveness of medication are some risk factors that lower MA among individuals with depression, regardless of comorbidities [34]. Furthermore, multimorbid individuals were found to have 2.13 times higher risk of depression due to health concerns and reduced quality of life from disability and pain [35]. Several studies have consistently reported the effect of SMC on depressive symptoms, the psychological burden associated with perceived decline in cognitive functions leading to depression among older adults [27, 36]. Prevalence of depression among hypertensive older adults was 57.1% [37], and those hypertensive patients with multimorbidity had significantly higher rates of depression [38].

On the other hand, the WHO has identified social support as a health system factor of MA among chronic disease patients [39]. A study of MA patterns in chronic disease patients identified social support, in particular perceived social support, as a key predictor of MA [40]. Hypertensive patients who received social support from family, friends, and healthcare providers have been shown to actively maintain treatment in the face of physical, social, and economic vulnerabilities, enabling higher levels of MA [41]. Upon perceiving memory declines, older adults with adequate social support are more likely to seek early intervention through screening and counseling [42]. Moreover, evidence on the positive effects of social support on the MA of hypertensive patients [43] suggest the importance of social support in improving MA in older adults with hypertension.

Most of the previous literature exploring factors related to MA in older adults with chronic diseases have focused on the population with a single chronic disease or a specific condition. Therefore, the current evidence does not reflect the needs of the aging population with multiple morbidities. Furthermore, studies have failed to identify the gaps of long-standing policies for chronic disease management, which have been largely ineffective in improving MA in older adults with polypharmacy. Previous literature has identified SMC [31], depression [33, 34], and social support [39–43] as individually associated with MA. However, exploratory studies on how SMC, depression, and social support interrelate with and influence MA are lacking, especially among hypertensive older adults with multimorbidity.

The purpose of this study is to determine the level of MA and identify the mediating effects of depression and social support in the relationship between SMC and MA among hypertensive older adults with multimorbidity residing in the community. Identification of the relationships among comprehensive geriatric factors associated with MA will provide preliminary evidence for development of customized care for improving MA at the primary care settings.

## Methods

### Study design and participants

This study is a descriptive cross-sectional investigation. It was conducted to identify the mediating effect of depression and social support on the effect of SMC on MA among hypertensive community-dwelling older adults with multimorbidity.

Study participants come from a convenience sample of older adults utilizing services across three community senior centers providing various physical, emotional and cognitive activities to older adults in City G. The inclusion criteria for participation include those hypertensive patients who are 65 years or older, with two or more chronic diseases including hypertension, and are taking prescribed medication for chronic diseases. Those who were unable to verbally communicate and/or make voluntary decisions due to neurological disorders or dementia were excluded.

The sample size was calculated using the G*Power 3.1.9.4 program. The effect size was set at a significance level of 0.05, a medium effect size of 0.15, and a power of 0.95. Therefore, the estimated sample size for this study was 184. Considering a dropout rate of 10%, a survey was conducted on 204 people. Among the 204 older adults responded to the survey, the final analytic sample included 195 individuals, excluding 9 individuals who gave incomplete responses to the survey. Consequently, 83,61, and 51 older adults from the each of the three community senior centers respectively participated in the survey.

### Data collection

Data collection was conducted from January to March of 2018, by two research assistants who received a 1-hour training session on the purpose of study, data collection methodologies, communication skills with older participants, and survey response collection. After receiving written consent to participate from participants, the research assistants collected survey data. The survey was conducted via a face-to-face survey, where research assistants filled out the paper survey responses on behalf of the older adult in a separate space. On average, participants took approximately 15 to 20 min to complete the survey. At this time, the research assistant read the questionnaire while monitoring the participant’s condition and the research assistant recorded the participant’s responses to the survey questions. Collected data was managed in compliance with IRB guidelines and all research materials were stored in a locked cabinet.

The study was approved by the Institutional Review Board (IRB) at Chonnam National University (IRB No. 1040198-171204-HR-088- 02).

### Measures

#### Subjective memory complaints

Subjective memory complaints was measured using Youn and colleagues’ Subjective Memory Complaints Questionnaire (SMCQ) [44]. The SMCQ, which assesses the severity of subjective memory loss, consists of a total of 14 items, including 3 items measuring subjective evaluation of overall memory impairment and 11 items which measure subjective memory impairments experienced in daily life. Each item is scored 0 for ‘no’ and 1 for ‘yes’, resulting in a measure range of 0–14, higher scores representing higher severity of subjective memory complaints. As for reliability, Cronbach’s α was 0.86 at the time of development [44], and 0.83 in the sample of this study.

#### Depression

Depression is measured by the Geriatric Depression Scale Short Form Korean Version (GDSSF-K) [45], which is translated from Sheikh and Yesavage’s Depression Scale Short-Form [46]. The GDSSF-K is consisted of 15 items, scored with 0 for ‘no’ and 1 for ‘yes’. Higher scores represent more severe degree of depressive symptoms. Reliability of the GDSSF-K was acceptable with Cronbach‘s α of 0.88 both upon development [45] and in this study’s sample.

#### Social support

Social support was measured using Park’s scale for perceived social support [47]. The scale is consisted of 7 items of emotional support, 7 items of informational support, 6 items of instrumental support/material support, and 6 items of evaluative support. Each item is rated on a 5-point Likert scale of ‘not at all’ (1 point), ‘to ‘very much’ (5 points). The total score ranges from 25 to 125, with higher scores indicating higher levels of social support. As for reliability, Cronbach’s α was.95 at the time of development [47], and Cronbach’s α was.97 in this study.

#### Medication adherence

MA was measured using Morisky and colleagues’ Morisky Medication Adherence Scale (MMAS-8) [48], which was translated into Korean [49]. The measure consists of 8 items, for which items 1–7 are scored 0 for ‘no’ and 1 for ‘yes’ excluding item 5 which scoring is reversed (0 for ‘yes’ and 1 for ‘no’). Item 8 is scored on a 4-point scale with ‘always’ (0 points), ‘often’ (0.25 points), ‘sometimes’ (0.5 points), ‘rarely’ (0.75 points), and ‘never’ (1 point). The MMAS-8 is rated on a scale of 0–8 with scores below 6 suggesting ‘medication non-adherence’, and scores 6 and above for ‘medication adherence’. Higher scores represent better adherence to prescribed medication. Cronbach’s α during development was.83 [49] and.74 in this study.

Participants’ characteristics.

This questionnaire included four items on sociodemographic characteristics and seven items on health-related characteristics. Sociodemographic characteristics included gender, age, education, living arrangement. Age was collected as a continuous variable and categorized into 65–74 years, 75–84 years, and 85 years or older. Education level was categorized into less than elementary school, middle school, high school, and college graduates, and living arrangement was categorized into living alone and living with others. Also, health-related characteristics include current alcohol consumption, current smoking, exercise in past 1 month, insomnia, healthcare accessibility, awareness of prescribed drugs and side effects of drugs. Current smoking was measured by through a ‘yes’ or ‘no’ question and previous smokers were defined as non-smokers. Current drinking was measured by whether respondent consumed alcohol within the previous month. Physical activity participation was defined as participating in at least 30 min of exercise, five or more times a week. Healthcare access was categorized into “very good”, “good”, and “not good”, and finally awareness of prescribed drugs and side effects were measured with a binary measure of ‘yes’ or ‘no’.

### Data analysis

The collected data was analyzed using IBM SPSS 26.0 and STATA MP 17.0 version. Descriptive statistics of frequencies, means, and standard deviations were analyzed for participants’ demographic and health-related characteristics, SMC, depression, social support, and MA. Bivariate analyses using Chi-squared tests, t-tests, and ANOVA were conducted to identify differences in SMC, depression, social support, and MA according to sociodemographic characteristics of participants. Pearson’s correlation coefficient was calculated to assess correlation among key variables of SMC, depression, social support, and MA. The PROCESS macro v4.2 [Model 6] program was used to analyze the mediating effect of depression and social support in the relationship between the subjects’ SMC and MA. The number of samples was 5,000 and analyzed with a 95% confidence interval. The statistical significance of the mediating effect was analyzed using the bootstrap method, using 5000 bootstrap samples analyzed with a 95% confidence interval.

## Results

### Sample characteristics and medication adherence according to demographic and health-related characteristics

Table 1 shows the sample characteristics and MA according to sociodemographic and health-related characteristics of participants. Of 195 participants, the average age was 75.5 (± 5.20), 73.9% were male, 42.1% were high school graduates, and 70.8% were living with someone else. As for health behaviors, drinking and smoking behaviors were observed in 28.2% and 5.1% of the sample respectively, and 91.3% of the sample reported to participate in physical activities. 61.5% reported good access to healthcare facilities and 35.4% reported insomnia. Awareness of prescribed drugs was present among 92.8% of participants while awareness of side effects was present in 59.0%.

**Table 1:** Sample characteristics and medication adherence according to demographic and health-related characteristics (*N* = 195)

Characteristics	Categories	N (%)/Mean ± SD	Medication Adherence	
			Mean ± SD	t/F(p-value)
Demographic				
Gender	Male	144(73.9)	6.82 ± 1.57	1.286(0.200)
	Female	51(26.1)	6.48 ± 1.75	
Age		75.54 ± 5.20		
	65–74	90(46.2)	6.71 ± 1.78	0.140(0.873)
	75–84	94(48.2)	6.74 ± 1.49	
	≥ 85	11(5.6)	6.98 ± 1.51	
Education	≤Elementary school	24(12.3)	6.64 ± 1.72	0.170(0.915)
	Middle school	38(19.5)	6.85 ± 1.68	
	High School	82(42.1)	6.67 ± 1.56	
	≥College	51(26.2)	6.81 ± 1.68	
Living arrangement	Alone	27(29.2)	6.57 ± 1.55	-0.941(0.348)
	Living with others	138(70.8)	6.81 ± 1.65	
Health-related characteristics				
Current alcohol consumption	Yes	55(28.2)	6.45 ± 1.95	-1.572(0.118)
	No	140(71.8)	6.85 ± 1.47	
Current smoking	Yes	10(5.1)	6.23 ± 2.10	-1.022(0.308)
	No	185(94.9)	6.76 ± 1.60	
Exercise (in past 1month)	Yes	178(91.3)	6.75 ± 1.66	0.431(0.667)
	No	17(8.7)	6.57 ± 1.11	
Insomnia	Yes	69(35.4)	6.32 ± 1.77	-2.698(0.008)
	No	126(64.6)	6.96 ± 1.49	
Healthcare accessibility	Very good	59(30.3)	7.00 ± 1.20	2.750(0.006)
	Good	120(61.5)	6.71 ± 1.74	
	Not good	16(8.2)	5.94 ± 1.87	
Awareness of prescribed drugs	Yes	181(92.8)	6.85 ± 1.51	3.716(< 0.001)
	No	14(7.2)	5.23 ± 2.25	
Awareness of side effects of drugs	Yes	115(59.0)	7.05 ± 1.23	3.275(< 0.001)
	No	80(41.0)	6.23 ± 1.98	

Bivariate analyses of MA according to participants’ sociodemographic and health-related characteristics revealed significant differences for presence of insomnia (t=-2.698, *p* =.008), healthcare access (F = 2.750, *p* =.006), awareness of prescribed drugs (t = 3.716, *p* <.001), and awareness of side effects (t = 3.275, *p* <.001). Average MMAS-8 scores for participants without insomnia was 6.96, whereas scores for participants with insomnia was 6.32. Better access to healthcare facilities was associated with higher MA. Those who responded that healthcare access was ‘very good’ had an average MMAS-8 score of 7.00, whereas those who responded access was ‘poor’ had an average score of 5.94. The average level of MA was 6.85 for those who were aware of their prescribed medication, but 5.23 for those who were unaware. The average MMAS-8 score for those who were aware of the side effects of the prescribed drugs was 7.05, whereas the average score for those unaware of side effects was 6.23.

### Descriptive statistics and correlations among the variables in the study

The descriptive statistics and correlations among SMC, depression, social support, MA, number of chronic diseases, and number of medications taken are presented in Table 2. The mean score for SMC and depression was 2.39 (± 2.56) and 3.49 (± 3.48) respectively. Average social support score was 91.99 (± 19.47) and average MA score was 6.74 (± 1.62). Participants of the study had an average of 2.72 (± 1.05) chronic diseases and were taking an average of 4.51 (± 3.74) medications. Average number of chronic diseases among participants was 2.72 (± 1.05), and average number of medications taken was 4.51 (± 3.74).

**Table 2:** Descriptive statistics and correlations among the variables (*N* = 195)

Variables	Mean ± SD	Min	Max	Range	Correlation matrix					
					1	2	3	4	5	6
1. SMC	2.39 ± 2.56	0	14	0–14	1					
2. Depression	3.49 ± 3.48	0	15	0–15	0.30^***^	1				
3. Social support	91.99 ± 19.47	50	125	25–125	-0.26^***^	-0.43^***^	1			
4. MA	6.74 ± 1.62	1.25	8	0 ~ 8	-0.24^***^	-0.34^***^	0.25^***^	1		
5. No. of chronic diseases	2.72 ± 1.05	2	10	2~	0.08	0.10	-0.15^**^	-0.17	1	
6. No. of prescription medications	4.51 ± 3.74	1	30	1~	0.02	0.08	0.08	-0.10	0.04	1

Abbreviations: SMC, subjective memory complaints; MA, medication adherence; No. of, number of

*p <.05, **p <.01, ***p <.001

Correlation analyses showed SMC were negatively correlated with social support (*r* = -.26, *p* <.001) and MA (*r*=-.24, *p* <.001) but positively correlated with depression (*r* =.30, *p* <.001). Furthermore, depression was negatively correlated with social support (*r*=-.43, *p* <.001) and MA (*r*=-.34, *p* <.001). Social support had a positive correlation with MA (*r* =.25, *p* <.001) and negative correlation with the number of chronic diseases (*r*=-.15, *p* =.04*).*

### Multiple mediation analyses

**Fig. 1:**
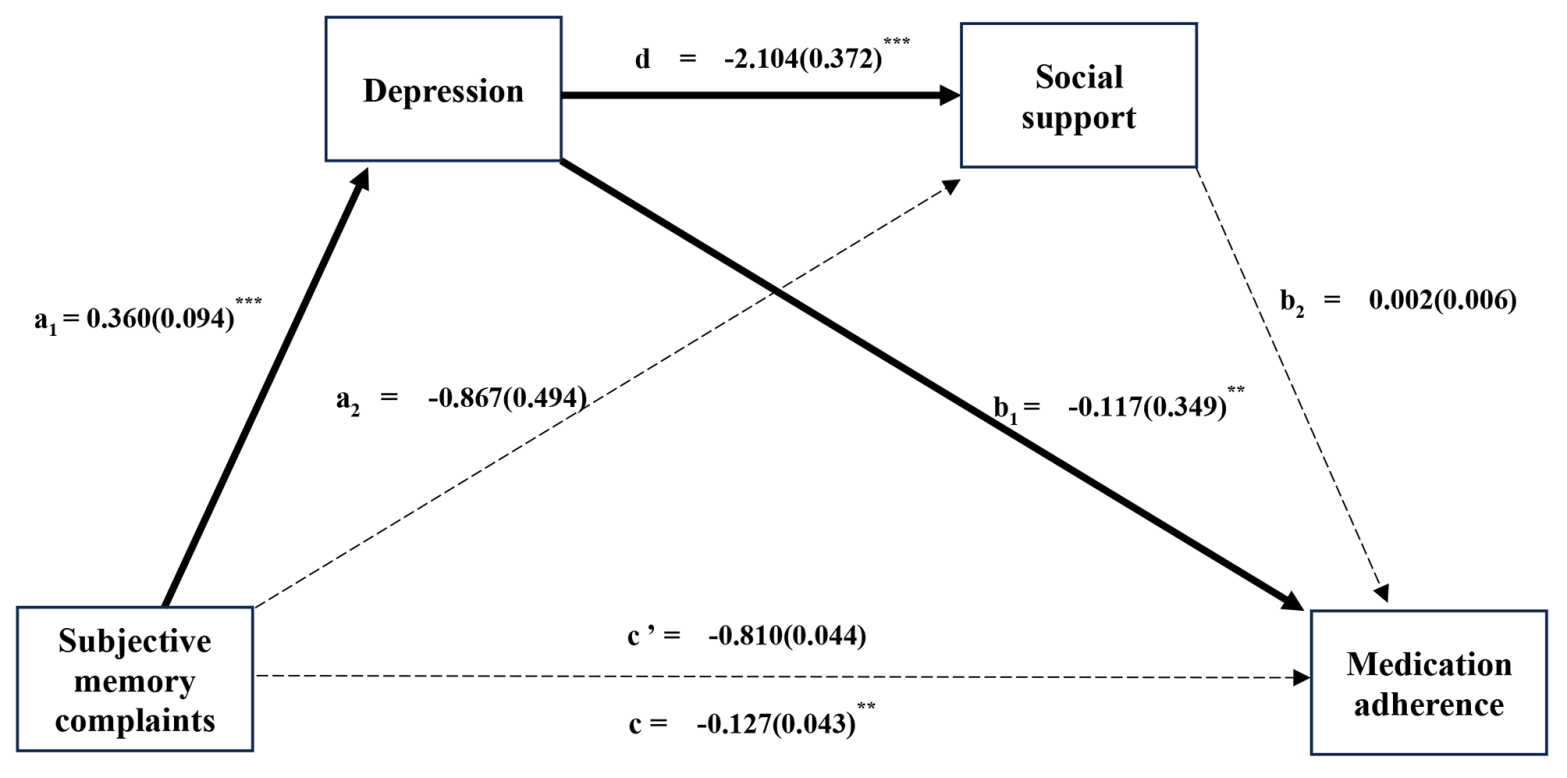
Results of the multiple mediation analyses, controlling for insomnia, healthcare accessibility, awareness of prescribed drugs and side effects of drugs and number of chronic diseases (*N* = 195). The parameter estimates were presented as B (SE). a_1_ = direct effect of subjective memory complaints on depression; a_2_ = direct effect of subjective memory complaints on social support; b_1_ = direct effect of depression on medication adherence; b_2_ = direct effect of social support on medication adherence; c’ = direct effect of subjective memory complaints on medication adherence; c = total effect of subjective memory complaints on medication adherence. *p <.05, **p <.01, ***p <.001

Results of the multiple mediation analyses are shown in Fig. 1; Table 3. While the direct effect of SMC on MA was not statistically significant, the direct effect on depression (B = 0.360, *p* <.001) was significant. The direct effects of depression on MA (B= -0.117, *p* =.001) and social support (B= -2.104, SE: 0.372, *p* <.001) were both statistically significant. However, neither the direct effect of SMC on social support, nor the effect of social support on MA were statistically significant.

**Table 3:** Indirect effects of SMC on MA mediated by depression and social support (*N* = 195)

Effect	B	SE	95% CI	
			LLCI	ULCI
Total indirect effect of SMC on MA	-0.046	0.020	-0.090	-0.011
SMC → Depression →MA	-0.042	0.020	-0.085	-0.008
SMC→ Social support → MA	-0.002	0.007	-0.019	0.012
SMC → Depression → Social support → MA	-0.002	0.006	-0.014	0.009

Abbreviations: SMC, subjective memory complaints; MA, medication adherence

Bootstrap sample size = 5000; *CI* confidence interval, *SE* standard error, LLCI lower limit confidence interval, ULCI upper limit confidence interval

The total effect of SMC on MA was statistically significant (B = -0.127, *p* <.01), and indirect effect of SMC on MA through depression was also significant (B= -0.042, *p* <.01).

## Discussion

This study was conducted to examine the mediating effects of depression and social support on the relationship between SMC and MA in community-dwelling, multimorbid hypertensive older adults.

In this study, MA was moderate with a mean score of 6.74, and the proportion of non-adherence indicated by a MA score of less than 6 was low at 27.7%. This may be attributed to the sample characteristics, consisting of participants recruited from senior community centers, who have been actively utilizing various leisure and health programs and health and welfare counseling services in the community. However, previous studies on MA among hypertensive older adults utilizing the senior welfare centers have shown higher rates of non-adherence at 41% [50]. Our results were also of contrast from meta-analysis of non-adherence of multimorbid patients which found a non-adherence rate of 42.6% [13]. Such differences can be attributed to the characteristics of the sample. Foley and colleagues observed MA in a sample of multimorbid patients, unrestricted to older adults and comorbidity of hypertension [13]. However, the study by Park and colleagues [50] was conducted in 2008–2009 among older adults with hypertension only. More than a decade has passed since Park’s study, since then baby boomers with relatively higher educational attainment and more active participation in health management have entered into older adulthood. The lower rates of non-adherence in our study may be attributed to such differences in sample characteristics.

Bivariate analyses of differences in MA by sociodemographic and health factors revealed that insomnia was significantly associated with MA. Insomnia among older adults is associated with various cognitive functions, especially that of SMC [51]. Furthermore, Lin and colleagues describe that nighttime insomnia and consequent excessive daytime sleepiness decreased MA in older adults [52]. While such findings are consistent with the results of our study, direct comparison is limited due to lack of studies investigating effect of insomnia on the MA of hypertensive patients with multimorbidity. Nonetheless, insomnia is a growing concern in the aging population, for which hypertension and intake of antihypertensive medications are risk factors [53]. To improve MA among older adults with hypertension and multimorbidity, it is necessary to assess insomnia and to explore strategies to alleviate insomnia.

Significant differences in MA were observed based on awareness of medications and its side effects. Successful MA among older adults require an awareness of the prescribed medications [20]. Awareness of medication name, purpose, and side effects have been found to be significantly associated with MA [54]. However, multimorbid patients with increasing numbers of medications may face difficulties in identifying the different intake directions associated with each medication [31]. Furthermore, polypharmacy, a common phenomenon among older adults with chronic conditions, has been associated with medication non-adherence, adverse drug effects, drug-drug interactions, functional decline, and increased risk of multimorbidity in older adults [55]. Polypharmacy in multimorbid older adults can lead to medication errors, adverse drug reactions, falls and dizziness [55]. Therefore, health care providers on the frontline of medication management of older patients should seek to improve patients’ awareness of prescribed medications and adverse drug reactions and develop medication management programs accordingly.

In this study, subjects showed significant differences in MA based on access to healthcare. Previous literature shows the importance of interactions with healthcare providers for successful MA [20], and that insufficient communication can contribute to medication non-adherence [56]. Furthermore, our results were aligned with those of a qualitative investigation of factors associated with MA in patients with hypertension identified frequency of physician visits and interactions with healthcare providers as indicators of MA [57].

Our results showed SMC was significantly inversely correlated with MA, indicating that as SMC increased, MA decreased. While the direct effect of SMC on MA was not statistically significant, the total effect was statistically significant, suggesting the importance of subjective memory amidst the increasing number of chronic diseases and medication complexity in this sample. On the other hand, prospective memory, the memory of tasks to be performed in the future, such as remembering to take medication at a certain time, has been reported to be an important factor in MA [58]. Individuals reporting SMC often report difficulties performing time-based prospective memory tasks [59], suggesting the significance of SMC on MA. Moreover, the proportion of older adults who seek help for SMC is very low [42]. Therefore, it is necessary to identify and screen for SMC upon assessment of MA for hypertensive patients with multimorbidity.

Correlation between SMC and depression was significantly positive while depression and MA was significantly negatively correlated in our study. Also, the direct effects of SMC on depression and depression on MA, and the mediating effect of depression on the effect of SMC on MA were all statistically significant. Our results are aligned with that of Steinberg and colleagues [27] which reported SMC was associated with stress, depression and anxiety among community-dwelling older adults [27](Steinberg et al., 2013)(Steinberg et al., 2013)(Steinberg et al., 2013)(Steinberg et al., 2013) [27](Steinberg et al., 2013)(Steinberg et al., 2013) [27](Steinberg et al., 2013)(Steinberg et al., 2013)(Steinberg et al., 2013) [27](Steinberg et al., 2013)(Steinberg et al., 2013)(Steinberg et al., 2013)(Steinberg et al., 2013)(Steinberg et al., 2013)(Steinberg et al., 2013)(Steinberg et al., 2013)(Steinberg et al., 2013)(Steinberg et al., 2013)(Steinberg et al., 2013) [27](Steinberg et al., 2013) and that of Zandi [36] who found depression was significantly higher among older adults with SMC. Furthermore, Branin’s study [33] showed that memory anxiety and depression were major predictors of MA among older adults, and Grenard and colleagues [34] conducted a meta-analysis of depression and MA in the treatment of chronic diseases, and found that the probability of medication nonadherence was 1.76 times higher in the presence of depression than in the absence of depressive symptoms, supporting the results of this study. This suggests that interventions for cognitive functions such as memory, as well as psychosocial interventions for depression, should be combined to increase MA in older adults with hypertension [60]. Therefore, health professionals at the primary health care settings should continuously monitor for depression in older adults with hypertension to prevent against medication non-adherence [37].

In this study, SMC was significantly inversely correlated with social support, and social support was significantly positively correlated with MA. On the other hand, neither the mediating effect of social support on the relationship between SMC and MA, nor the direct effect of social support on MA were statistically significant. While the direction of correlation was relevant, social support as an independent factor of MA was not statistically significant. Such results were contrary to previous studies which observed that social support provided by family and friends to hypertensive patients has a significant effect on MA [40, 61, 62]. Such difference can be attributed to the multidimensional nature of social support, such as its structural and functional aspects. Whereas structural social support is measured through the number of social relationships and frequency of contact, functional social support focuses on the functional support received from the formation of relationships, through reciprocating information and emotional support [63]. The difference in the impact of social support on MA may be due to the difference in the dimension of social support measured. The social support measured in this study was functional social support, while previous literatures have mostly focused on structural support measures. Moreover, unlike previous studies, which had a relatively high proportion of female participants, the majority of the participants in this study were male. There are gender differences in social support and social networks throughout the aging process [64]; in general, women have more friends in the community, receive more social support, and have more diverse social networks than men [64]. In general, women have also been shown to be more sensitive to the needs of others, more prone to provide social support to others and also have a higher need for social support themselves [65]. Therefore, the high proportion of male older adults in this study, who have relatively low needs and dependence on social support, may explain the discrepancy with previous studies. Future research should utilize social support instruments that consider the multidimensional aspects of social support and also take into account the differences in sample characteristics.

In this study, depression was significantly inversely correlated with social support, indicating that higher social support was associated with lower depression among hypertensive older adults with multiple comorbidities. However, the multiple mediating effect of depression and social support on the relationship between SMC and MA was not statistically significant. Such findings may be due to the predominance of male participants in this study. Gender differences in depression are one of the most well-known phenomena in psychiatry and psychology, with the onset and prevalence of depression being lower in men than in women [66, 67]. Likewise, the magnitude and dependence of social support is lower in men than in women [64, 65]. Further exploration is required to determine whether the lack of significant multiple mediation effect is due to the sample characteristics, or whether social support functions as a moderator rather than a mediator.

In light of the above, the significance of this study is as follows. First, unlike previous studies, this study focused on hypertensive elderly with multimorbidity rather than hypertension alone, exhibiting the importance of medication management for multimorbidity due to polypharmacy as the number of diseases increases. Second, we measure cognitive decline using SMC, which is more closely associated with the older adults experience of MA in the daily lives of older adults. Finally, by demonstrating that depression mediates the effect of SMC on MA in older adults with hypertension, this study suggests the significance of measuring depressive symptoms through comprehensive health and developing strategies to prevent and manage depression to improve MA in older adults. The implications of our findings can be applied to building appropriate medication-based disease management for hypertensive older adults with multimorbidity to contribute to preventing complications and improving quality of life.

Nevertheless, the limitations of this study are as follows. The recruitment of the study sample of hypertensive older adults with multimorbidity was from a convenience sample of older adults who frequently utilize various health and welfare programs across three senior welfare centers located in a metropolitan area of Korea. Therefore, future studies on the effect of SMC on MA should involve representative samples to generalize the results of the study.

## Conclusion

This study was conducted to assess the level of MA among community-dwelling hypertensive older adults with multimorbidity, to determine the correlation between SMC, depression, social support, and MA, and to test the mediating effects of depression and social support on the relationship between SMC and MA. Results showed that the MA level of hypertensive elderly with multiple comorbidities was moderate, and lower SMC, lower depressive symptoms, and higher social support was associated with better MA. In particular, the mediating effect of depression on the SMC and MA calls for a need to screen for and manage depression in the process of medication management in the community. In conclusion, to prevent complications and improve quality of life through MA among older adults with multimorbid hypertension, health care providers in the community should conduct comprehensive health assessments to assess not only biological and physical indicators associated with medications but also psychosocial factors related to MA.

## Data Availability

The datasets generated and/or analyzed during the current study are not publicly available due to the policy of the CNU IRB, which does not allow the opening and sharing of research data with any third party, but are available from the corresponding author upon reasonable request.

## Abbreviations

MA: Medication Adherence
SMC: Subjective Memory Complaints
WHO: World Health Organization
